# Acid Sphingomyelinase Serum Activity Predicts Mortality in Intensive Care Unit Patients after Systemic Inflammation: A Prospective Cohort Study

**DOI:** 10.1371/journal.pone.0112323

**Published:** 2014-11-10

**Authors:** Matthias Kott, Gunnar Elke, Maike Reinicke, Supandi Winoto-Morbach, Dirk Schädler, Günther Zick, Inéz Frerichs, Norbert Weiler, Stefan Schütze

**Affiliations:** 1 Department of Anesthesiology and Intensive Care Medicine, University Medical Center Schleswig-Holstein, Campus Kiel, Kiel, Germany; 2 Institute of Immunology, University Medical Center Schleswig-Holstein, Campus Kiel, Kiel, Germany; University of Florida College of Medicine, United States of America

## Abstract

**Introduction:**

Acid sphingomyelinase is involved in lipid signalling pathways and regulation of apoptosis by the generation of ceramide and plays an important role during the host response to infectious stimuli. It thus has the potential to be used as a novel diagnostic marker in the management of critically ill patients. The objective of our study was to evaluate acid sphingomyelinase serum activity (ASM) as a diagnostic and prognostic marker in a mixed intensive care unit population before, during, and after systemic inflammation.

**Methods:**

40 patients admitted to the intensive care unit at risk for developing systemic inflammation (defined as systemic inflammatory response syndrome *plus* a significant procalcitonin [PCT] increase) were included. ASM was analysed on ICU admission, before (*PCT_before_)*, during (*PCT_peak_*) and after (*PCT_low_)* onset of SIRS. Patients undergoing elective surgery served as control (N = 8). Receiver-operating characteristics curves were computed.

**Results:**

ASM significantly increased after surgery in the eight control patients. Patients from the intensive care unit had significantly higher ASM on admission than control patients after surgery. 19 out of 40 patients admitted to the intensive care unit developed systemic inflammation and 21 did not, with no differences in ASM between these two groups on admission. In patients with SIRS and PCT peak, ASM between admission and *PCT_before_* was not different, but further increased at *PCT_peak_* in non-survivors and was significantly higher at *PCT_low_* compared to survivors. Survivors exhibited decreased ASM at *PCT_peak_* and *PCT_low_*. Receiver operating curve analysis on discrimination of ICU mortality showed an area under the curve of 0.79 for ASM at *PCT_low_*.

**Conclusions:**

In summary, ASM was generally higher in patients admitted to the intensive care unit compared to patients undergoing uncomplicated surgery. ASM did not indicate onset of systemic inflammation. In contrast to PCT however, it remained high in non-surviving ICU patients after systemic inflammation.

## Introduction

Despite advances in critical care, systemic inflammatory response syndrome (SIRS) and sepsis syndrome with subsequent multi-organ failure still contribute to overall mortality in critically ill patients, equalling the number of deaths caused by acute myocardial infarction [1]. Besides specific treatment of the underlying cause of systemic inflammation, early diagnosis based on clinical findings and laboratory testing is of paramount importance to enable successful therapy [2]. The kinetics of biomarkers reflecting changes in the inflammatory condition can be helpful to identify patients at high risk for complications.

At present, procalcitonin (PCT) is regarded as the best available laboratory tool for the diagnosis of infection and systemic inflammation in combination with clinical symptoms [3], [4] and has been introduced as a variable in the diagnostic criteria for sepsis in the recently updated Surviving Sepsis Campaign guidelines [5]. PCT has the advantage of an earlier peak level upon infection, a better specificity and correlation to disease severity and clinical outcome as compared to routine biomarkers such as white blood cell count or C-reactive protein (CRP) [6]–[9].

Activation of acid sphingomyelinase, a C-type phosphodiesterase leads to generation of ceramide from biological inert sphingomyelin derived from the cell membrane. Generation of ceramide at the outer leaflet of cell membranes induces changes in composition and spatial arrangement in terms of forming ceramide-enriched membrane lipid rafts, leading to receptor clustering and apoptosis signalling and modification of the cellular response to stress stimuli [10], [11]. Clinical data concerning the potential role of acid sphingomyelinase serum activity (ASM) is infrequent. In a retrospective study of 12 patients with severe sepsis, Claus et al. showed that elevated ASM levels can be observed after the onset of sepsis, and that a further increase may be associated with worse outcome [12]. On the other hand, recent animal studies suggest a protective role of ASM secretion during the early host response as a first line of defence [13]. Due to its pathophysiological properties in the early host response, determination of ASM may also be used as an early diagnostic marker before the onset of systemic inflammation. The aim of our pilot study was to evaluate the role of ASM in a mixed intensive care unit (ICU) population at risk for the development of systemic inflammation. We hypothesized that ASM a) increases before the onset of systemic inflammation, and b) may be used as a prognostic marker of outcome.

## Materials and Methods

### Study design and patients

This single-center prospective cohort study was conducted in two surgical intensive care units of a tertiary medical center (University Medical Center Schleswig-Holstein, Campus Kiel, Kiel, Germany) with the approval of the local ethics committee of the Christian-Albrechts University Kiel, Germany. Written informed consent was obtained from each patient or the legal representative, respectively.

We prospectively included patients (age≥18 years) who were admitted to the ICU and deemed to be at high risk for the development of systemic inflammation (ICU group). High risk criteria were defined as the following ICU admission diagnoses: extended surgery, polytrauma, respiratory insufficiency, and patients with an expected length of ICU stay >3 days. Patients were evaluated on a daily basis for new onset of systemic inflammatory response syndrome (SIRS) fulfilling two or more of the defined clinical criteria according to the ACCP/SCCM recommendations [14] *plus* an increase in PCT. This PCT peak was defined as a two-fold increase in PCT concentration compared to the value of the preceding day and exceeding a minimum of >2 ng/ml. Exclusion criteria were diseases associated with hyperprocalcitoninaemia like small-cell lung cancer or C-cell carcinoma and administration of PCT inducing agents (Anti-Thymocyte globulin or OKT3 antibodies). Events with PCT elevations following re-operation during the ICU stay and patients receiving ASM inhibiting medications (amlodipin, sertralin, imipramin, desipramin, or steroids) were also excluded [15]. ASM was consecutively analysed at four time points: I), on ICU admission, II), the day before new onset of SIRS in combination with a PCT peak were detected (*PCT_before_*), III), the first day of new onset of SIRS and a PCT peak (*PCT_peak_*), and IV), on the first day when the PCT level was below 0.5 ng/ml or declined to a nadir with less than 30% of the *PCT_peak_* concentration (*PCT_low_*).

Patients undergoing uncomplicated elective major abdominal surgery were used as a control group to assess the peri-operative kinetics of ASM. In these patients, ASM was also determined before surgery to determine the influence of the surgical trauma on ASM. Serum samples for the analysis of ASM were collected prospectively on a daily basis and stored at −20°Celsius. ASM on the day of ICU admission was analysed in all included patients but due to financial restraints consecutive measurements of ASM (from *PCT_before_* until patients’ discharge or death) were performed only in those patients with a PCT peak.

### Data collection

We collected baseline characteristics of the patients including demographic information, comorbidities and type of surgery. Severity of illness was determined by calculating the Simplified Acute and Physiology Score II (SAPS II) [16], Therapeutic Intervention Scoring System (TISS) [17] and a modified Sequential Organ Failure Assessment Score (mSOFA) [18] (excluding central nervous system). Serum samples for the analysis of PCT and ASM (10 ml of either central venous or arterial blood) were prospectively collected on a daily basis in addition to routine laboratory including white blood cell count (WBC), lactate, C-reactive protein (CRP) serum levels. The patients’ management was left at the discretion of the attending ICU physician. Patients were followed up until discharge from the ICU or death.

### Measurements of ASM serum activity

Plasma was obtained and centrifuged at 3000 g for 5 minutes (Sorvall -Super TRI, Kendro Laboratory Products GmbH, Langenselbold, Germany) and stored at −20°C until assayed. Analytical determination of ASM depended on detection of radio-labeled [14C]-Phosphorycholin that was generated by [14C]-sphingomyelin cleavage in aequimolar amounts to ceramide [19]. Protein quantity was determined by bicinchoninacid (BCA)-assays. 300 µg of purified protein were used in a total volume of 10 µl per assay. 100 µl ASM buffer and 40 µl of [14C]-substrate were added and incubated for at least 2 hours at 37°C. Reaction was stopped by adding 750 µl chloroforme/methanol (2∶1) and 300 µl of destilled water. After 4 minutes of centrifugation by 14.000×g revolutions per minute, 300 µl of the upper aqueous phase were pipetted and filled into a scintillation test tube. 4 ml of scintillation fluid were added (Aquasafe 300 plus, Zinsser Analytic, Frankfurt), and β-count of radio-labeled [14C]-Phosphorycholin was measured (LS 6000LL, Beckman Coulter GmbH, Krefeld, Germany). ASM was calculated as pmol/ml•h.

### PCT measurements

PCT measurements were performed using a commercially available immunoluminometric assay (Elecsys BRAHMS PCT, BRAHMS-Diagnostica, Berlin, Germany) according to the manufacturer’s instructions via the automated Kryptor platform (BRAHMS AG, Hennigsdorf, Germany). The direct measuring range of the assay is from 0.02–100 ng/ml, with automated dilution extending the upper range to 1.000 ng/ml. The functional assay sensitivity is 0.06 ng/ml, and the sample volume needed is 50 µl.

### Statistical analysis

Means ± standard deviations (SD) or medians with interquartile ranges (IQR) are reported as appropriate. Differences in continuous variables between survivors and non-survivors were compared with the nonparametric Mann-Whitney test. Predictive values of serum ASM, PCT, CRP, WBC, body temperature and severity of illness measures regarding ICU mortality were evaluated. Discriminatory power (ability to distinguish between patients who die and those who survive) of laboratory tests and severity of illness scores were tested at all 3 time points to produce receiver operating characteristic (ROC) curves. The area under curve (AUC), with 95% confidence intervals (CI) and cut-offs for sensitivity and specificity were calculated in prediction of ICU mortality. A *P* value below 0.05 was considered statistically significant. Statistical analyses were performed with SPSS 17.0 (SPSS Inc., Chicago, IL, USA) and Prism 5 (GraphPad Software Inc., San Diego, CA, USA).

## Results

### Patient characteristics

**Figure 1:**
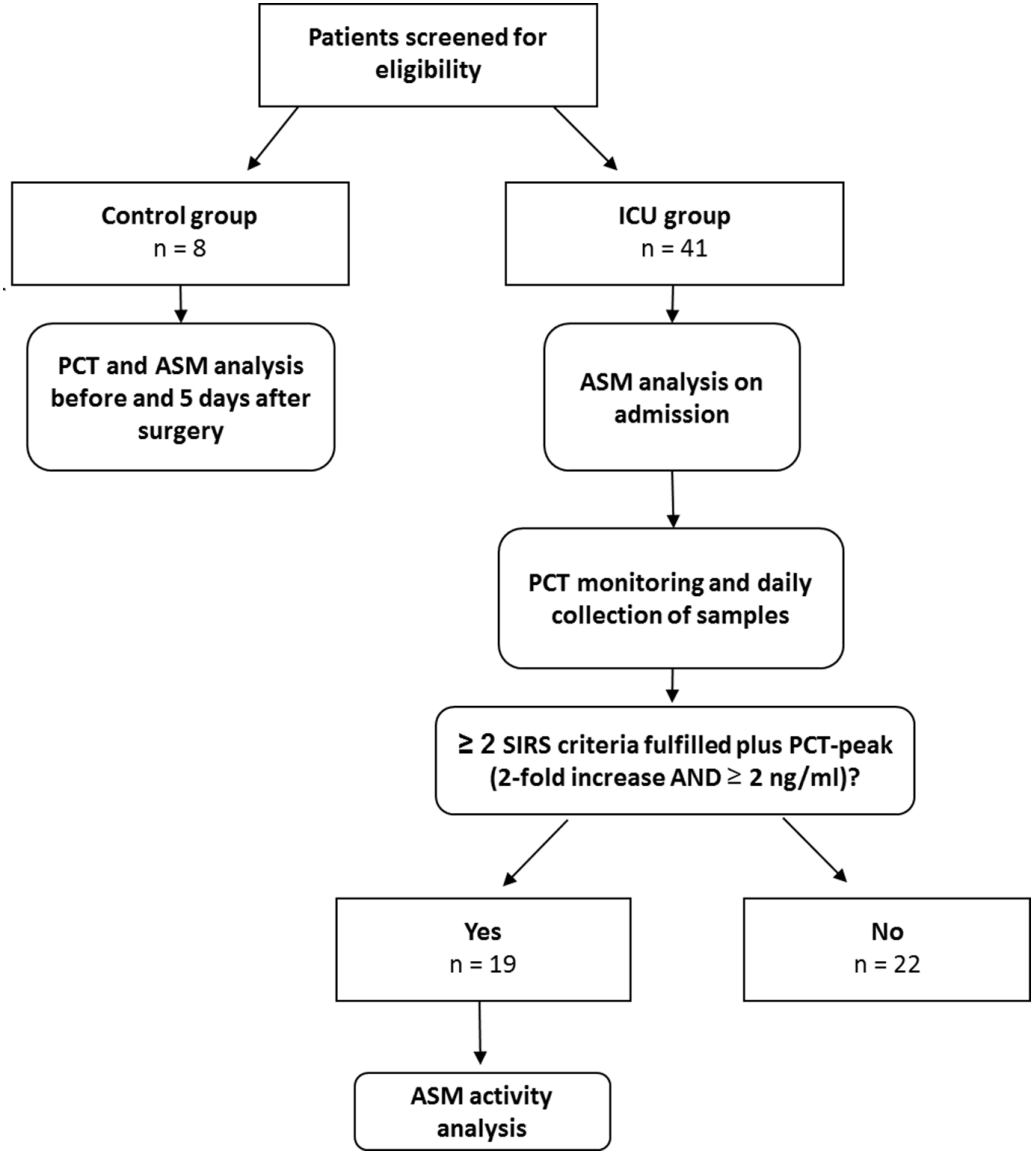
Study flow chart.

A study flow chart is given in Figure 1. A total of 48 patients were included during the study period of whom 8 patients undergoing uncomplicated major abdominal surgery served as the control group. 40 patients fulfilled high-risk criteria and were included in the ICU group. Finally, 19 patients in the ICU group had new onset of SIRS *plus* a PCT peak (one patient with two episodes). Table 1 shows patients’ baseline characteristics.

**Table 1:** Baseline patient characteristics and outcomes.

	Total		ICU Group		Control
		Total	SI	No SI	
**Number**	48	40	19	21	8
**Age,** median, years	69	69	69	69	65
**Gender,** male, n (%)	30 (62.5)	25 (63.4)	12 (63.2)	13 (66.6)	5 (62.5)
**Mortality,** n (%)	15 (31.2)	15 (36.6)	9 (47.4)	6 (27.3)	0
**Main diagnosis, n (%)**					
Abdominal surgery	22 (45.8)	14 (34.1)	8 (42.1)	6 (28.6)	8 (100)
Liver failure	1 (2.4)	1 (2.4)	1 (5.3)	-	-
Major bleeding	2 (4.9)	2 (4.9)	-	2 (9.5)	-
Major trauma	3 (7.3)	3 (7.3)	1 (5.3)	2 (9.5)	-
Mesenterial ischemia	2 (4.9)	2 (4.9)	-	2 (9.5)	-
Pancreatitis	2 (4.9)	2 (4.9)	2 (10.5)	-	-
Pneumonia	6 (14.6)	6 (14.6)	2 (10.5)	4 (19)	-
Soft tissue infection	3 (7.3)	3 (7.3)	-	3 (14.3)	-
Thoracic surgery	7 (17)	7 (17)	5 (26.3)	2 (9.5)	-

Systemic inflammation was defined as new onset of systemic inflammatory response syndrome plus a two-fold increase in PCT concentration compared to the value of the preceding day or exceeding a minimum of >2 ng/ml. PCT: procalcitonin, ICU: intensive care unit; SI: systemic inflammation.

### ASM, PCT and CRP levels in the control group

**Figure 2:**
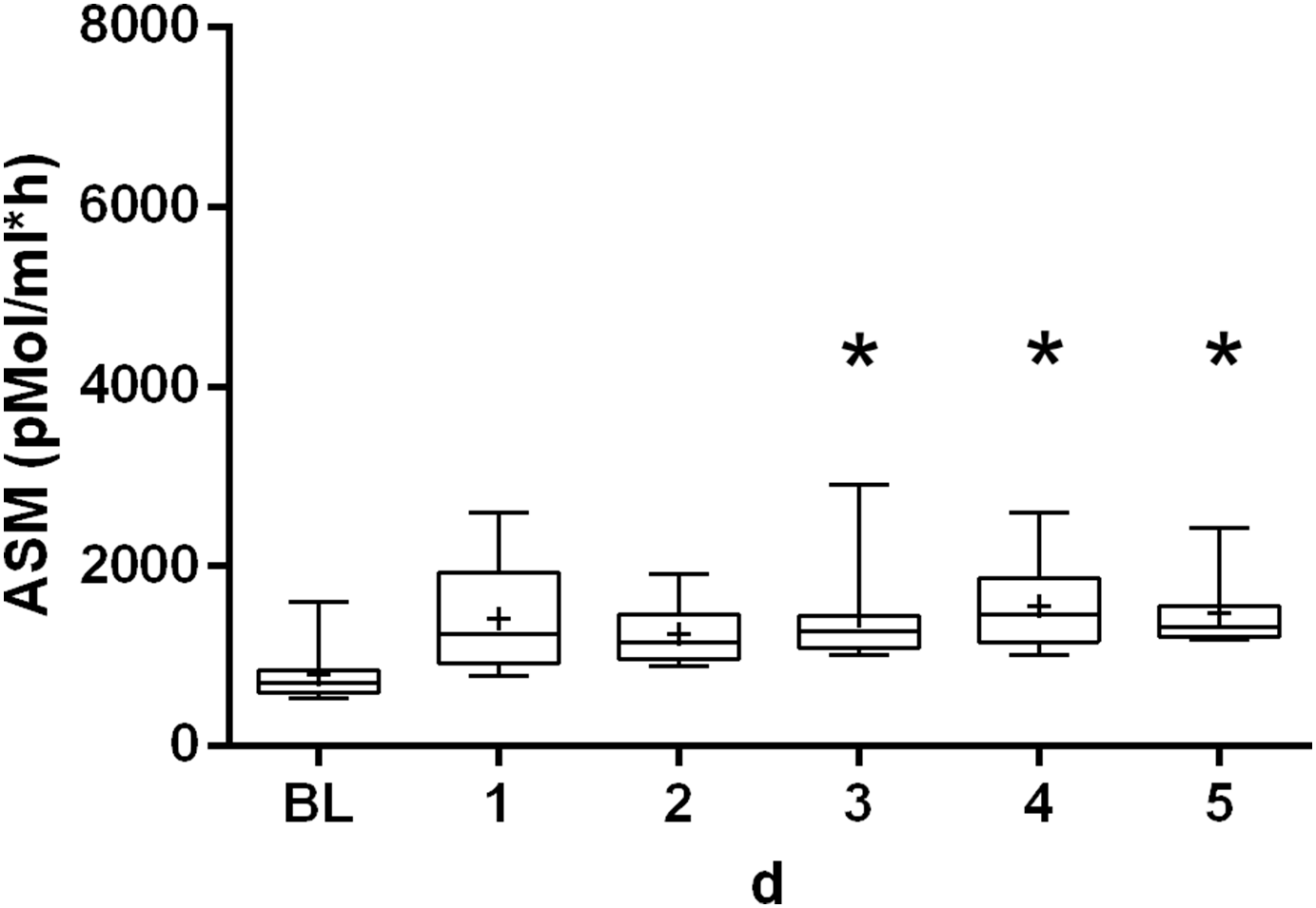
Kinetics of acid sphingomyelinase serum activity in the control group. The box plots display the minima/maxima (whiskers), 25%/75% percentile (box), median (−) and mean (+) values. Statistically significant differences (*P*<0.05) between baseline (BL) and post-operative values are marked with an asterisk (*). ASM: acid sphingomyelinase activity; BL: baseline (before surgery); d: days.

Figure 2 shows the pre- and post-operative kinetics of ASM in the control group. Median ASM measured before surgery was 709 pMol/ml•h (598–841 pMol/ml•h) and was significantly higher post-operatively (1320 pMol/ml•h; IQR: 1108–1565 pMol/ml•h). PCT and CRP both showed regular post-operative kinetics (data not shown).

### ASM, PCT, CRP and lactate levels in the ICU group

**Figure 3:**
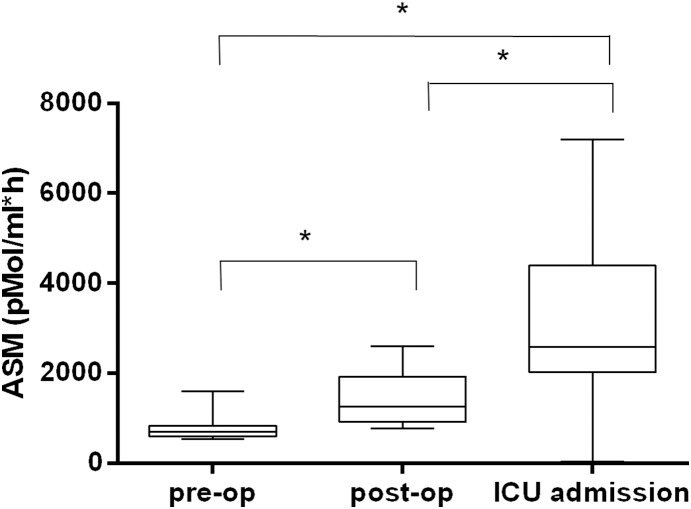
Acid sphingomyelinase serum activity in control and ICU patients. This figure indicates median pre- and post-operative acid sphingomyelinase serum activity of the control group and median acid sphingomyelinase serum activity of the ICU group on admission. Statistical significant differences between groups (*P*<0.05) are marked with an asterisk (*). The box plots display the minima/maxima (whiskers), 25%/75% percentile (box), and median (−) values. ASM: acid sphingomyelinase serum activity.

ASM, PCT, CRP, lactate levels and severity of illness measures at ICU admission are shown in Table 2. Median ASM upon ICU admission in the ICU group was significantly higher than both baseline and post-operative values in the control group (Figure 3). In 21 patients of the ICU group, no newly developed SIRS plus a PCT peak were observed and median ASM upon ICU admission was not different when compared to the other 19 patients with PCT peak (*P* = 0.8695). Table 3 summarizes the course of biomarker level in the 19 patients with new onset of SIRS plus a PCT peak. Median PCT level at *PCT_before_* was 0.46 ng/ml, increased markedly on *PCT_peak_* to 4.43 ng/ml and subsequently returned to nearly baseline values at *PCT_low_*. ASM showed no statistical significant differences between these three time points. Also, there was no difference in ASM in these patients between ICU admission and *PCT_before_* (*P* = 0.8995). There were no significant changes in CRP, lactate levels and severity of illness measures (table 3).

**Table 2:** Biomarker level and severity of illness measures of all study patients.

Variabe	ICU admission	ICU group		Control group
	(N = 40)	SI (N = 19)	No SI (N = 21)	(N = 8)
ASM (pMol/ml•h)	2593 (2021–4396)	2755 (1897–4396)	2529 (2041–4618)	709 (598–841)
PCT (ng/ml)	3.19 (1.03–8.15)	2.1 (0.75–4.88)	4.17 (1.57–10.38)	0.16 (0.12–0.23)
CRP (mg/l)	121 (35–274)	153 (12–235)	90 (35–304)	2.35 (0.97–25)
Lactate (mMol/l)	1.7 (0.9–2.5)	1.4 (0.8–2.6)	1.7 (1.1–2.5)	-
mSOFA score	7 (4–8)	7 (4–8)	6.5 (4–9)	-
SAPS II	46 (36–57)	46 (34–57)	48 (37–61)	-
TISS	19 (13–24)	14 (10–19)	22 (14–26)	-

In the ICU group, biomarker levels were measured in all patients at intensive care unit admission. For the control group, the pre-operative biomarker levels are displayed. Data are shown as median with interquartile ranges. Systemic inflammation was defined as new onset of systemic inflammatory response syndrome plus a two-fold increase in PCT concentration compared to the value of the preceding day or exceeding a minimum of >2 ng/ml. ASM: acid sphingomyelinase serum activity; CRP: C-reactive protein; mSOFA: modified Sequential Organ Failure Assessment score (excluding central nervous system); PCT: procalcitonin; SAPS II: Simplified Acute and Physiology Score II; SI: systemic inflammation; TISS: Therapeutic Intervention Scoring System.

**Table 3:** Biomarker level and severity of illness measures in patients with systemic inflammation (N = 19) according to PCT time points.

Variable	Time point	All (N = 19)	Survivor (N = 9)	Non-survivor (N = 10)	*P* Value*
**Procalcitonin**	*PCT_before_*	0.46 (0.29–2.08)	0.37 (0.19–1.38)	0.65 (0.42–2.78)	0.07
	*PCT_peak_*	4.43 (2.54–11.74)	4.14 (2.42–21.43)	4.43 (2.81–10.65)	>0.99
	*PCT_low_*	0.48 (0.43–1.09)	0.45 (0.41–0.72)	0.59 (0.44–1.12)	0.57
**ASM**	*PCT_before_*	2782 (1991–3903)	2777 (1835–3244)	2874 (2278–4528)	0.40
	*PCT_peak_*	3233 (1968–2907)	2501 (1953–3878)	3855 (1987–5122)	0.23
	*PCT_low_*	2261 (2063–2907)	2205 (1596–2496)	2611 (2235–4402)	***0.03***
**CRP**	*PCT_before_*	130 (87–220)	157 (74–210)	127 (80–233)	0.23
	*PCT_peak_*	145 (95–256)	124 (77–237)	182 (111–295)	0.25
	*PCT_low_*	98 (66–203)	70 (57–138)	141 (78–228)	***0.04***
**Lactate**	*PCT_before_*	1.6 (1.1–3.8)	1.6 (0.9–3.05)	1.8 (1.3–4.5)	0.62
	*PCT_peak_*	1.6 (1–3.2)	1.4 (1.1–3.2)	1.8 (1–3.9)	0.59
	*PCT_low_*	1.1 (0.9–1.6)	1.1 (0.8–1.6)	1.1 (0.9–2.3)	0.54
**mSOFA**	*PCT_before_*	7 (5–11)	7 (6–11)	7 (4–11)	0.67
	*PCT_peak_*	8 (5–11)	8 (6–12)	6 (3–11)	0.39
	*PCT_low_*	4 (2–6)	4 (3–7)	4 (1–6)	0.37
**SAPS II**	*PCT_before_*	48 (37–50)	46 (37–49)	49 (29–55)	0.54
	*PCT_peak_*	44 (37–51)	44 (40–46)	46 (30–58)	0.59
	*PCT_low_*	41 (35–48)	39 (32–43)	43 (38–53)	0.17
**TISS**	*PCT_before_*	21 (17–24)	22 (18–23)	20 (15–30)	0.88
	*PCT_peak_*	18 (15–23)	18 (15–23)	20 (8–24)	0.73
	*PCT_low_*	17 (6–23)	16 (9–19)	18 (0–26)	0.85

Time points were the day before the PCT peak (*PCT_before_*), the day of the PCT peak (*PCT_peak_*) and again lowered PCT (*PCT_low_*).

*P value for comparison of survivors vs. non-survivors. Systemic inflammation was defined as new onset of systemic inflammatory response syndrome plus a two-fold increase in PCT concentration compared to the value of the preceding day or exceeding a minimum of >2 ng/ml. ASM: acid sphingomyelinase serum activity, CRP: C-reactive protein concentration, mSOFA: modified Sequential Organ Failure Assessment Score (excluding central nervous system); PCT: procalcitonin; SAPS II: Simplified Acute and Physiology Score II; TISS: Therapeutic Intervention Scoring System.

### Survivors vs. non-survivors

**Figure 4:**
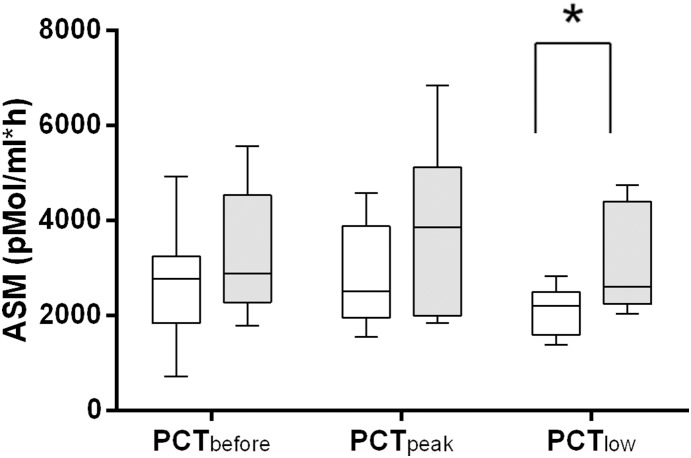
Acid sphingomyelinase serum activity in ICU patients with SIRS. Clear boxes represent survivors (n = 9) and grey boxes non-survivors (N = 10). Statistically significant difference (*P*<0.05) between survivors and non-survivors at *PCT_low_* (*) for acid sphingomyelinase serum. The box plots show the minima/maxima (whiskers), 25%/75% percentile (box), and median (−) values. ASM: acid sphingomyelinase serum activity.

Among the 19 patients with SIRS and a PCT peak, 9 patients (47.4%) died during their ICU stay as opposed to 6 of the 21 patients without PCT peak who died (27.3%). With *PCT_low_* as the baseline of ASM (equalling 100%), ASM increased only in non-survivors (by 34%), whereas in survivors a decline (by 10%) of ASM was detected even at *PCT_peak_*. ASM declined in all patients at *PCT_low_* (91% vs. 79%), but remained significantly higher in non-survivors when compared to survivors (*P* = 0.03; Table 3 and Figure 4).

### ROC analysis for ICU mortality in the ICU group

ROC analyses were performed at all four time points for all measured laboratory parameters. Only at *PCT_low_*, the areas under receiver operating curves (AUC) for ASM in prediction of ICU mortality were significantly different from 0.5 (AUC: 0.79, *P* = 0.03) with a sensitivity of 40% and specificity of 90% at an optimal cut-off value of 2098 pMol/ml•h, (likelihood ratio: 4). For CRP, the sensitivity was 60% and specificity 90% at an optimal cut-off value of 77.1 mg/L (Table 4).

**Table 4:** Receiver operating characteristics curve analysis for patients with systemic inflammation at *PCT_low_* (N = 19).

Variable	*P* Value	AUC	Optimal cut-off	Sensitivity (%)/Specifity (%)	LR
**ASM (pMol/ml**•**h)**	0.03	0.79	<2098	40/90	4
**PCT**	0.55	0.58	-	-	-
**CRP**	0.04	0.77	0.77	60/90	6
**Lactate**	0.52	0.58	-	-	-

At the two other time points, receiver operating characteristics curves were not significantly different from 0.5. Systemic inflammation was defined as new onset of systemic inflammatory response syndrome plus a two-fold increase in PCT concentration compared to the value of the preceding day or exceeding a minimum of >2 ng/ml. ASM: acid sphingomyelinase serum activity; AUC: area under the curve; LR: likelihood ratio; CRP: C-reactive protein; PCT: procalcitonin.

## Discussion

This prospective pilot cohort study evaluated the role of ASM as a potential early diagnostic and prognostic marker in a mixed ICU population at high risk of developing systemic inflammation. We found that ASM was generally increased in a mixed ICU population as compared to patients who underwent uncomplicated surgery. ASM was not found to be an early marker before the clinical onset of systemic inflammation as defined by fulfilling clinical SIRS criteria plus a PCT increase. However, ASM remained elevated in the presence of a low PCT level in non-surviving patients after systemic inflammation.

To our best knowledge, only two clinical studies have yet evaluated the role of ASM in patients with systemic inflammation. In a retrospective study of 12 severely septic patients, Claus et al. were able to show that sphingolytic activity was significantly increased compared to 13 healthy volunteers and that a further increase during the clinical course was proportional to the severity of illness (as indicated by a higher SOFA score on the day of maximal ASM) and paralleled by fatal outcome [12]. Drobnik et al. accordingly reported that ASM as reflected by the ceramide/sphingomyelin ratio was higher in septic patients compared to healthy controls and associated with a poor clinical outcome [20]. While both studies included patients with established sepsis, we evaluated for the first time the kinetics of ASM in ICU patients before systemic inflammation became clinically evident as defined by SIRS criteria plus a PCT increase. We evaluated the potential of ASM to serve as an early marker of inflammation as compared to established biomarkers such as PCT and CRP during the course of disease, and used a pragmatic approach including patients at high risk for developing systemic inflammation. In the 19 ICU patients who developed systemic inflammation during their ICU stay, there were no significant increases in ASM on the day before patients developed new SIRS plus a PCT peak elevation (*PCT_before_*). ASM levels were also not significantly different between patients with or without systemic inflammation upon ICU admission. Thus, ASM may not be a marker to indicate inflammation at an earlier stage than PCT.

In the control group, we observed a significant increase in median ASM after surgery parallel to a post-operative PCT and CRP increase. This increase reflects a “post-trauma” effect that is most likely explained by a lysosomal release of ASM resulting from tissue damage during surgery. However, the pre-operative and median post-operative ASM levels in the control group were still significantly lower as compared to the ICU patients.

**Figure 5:**
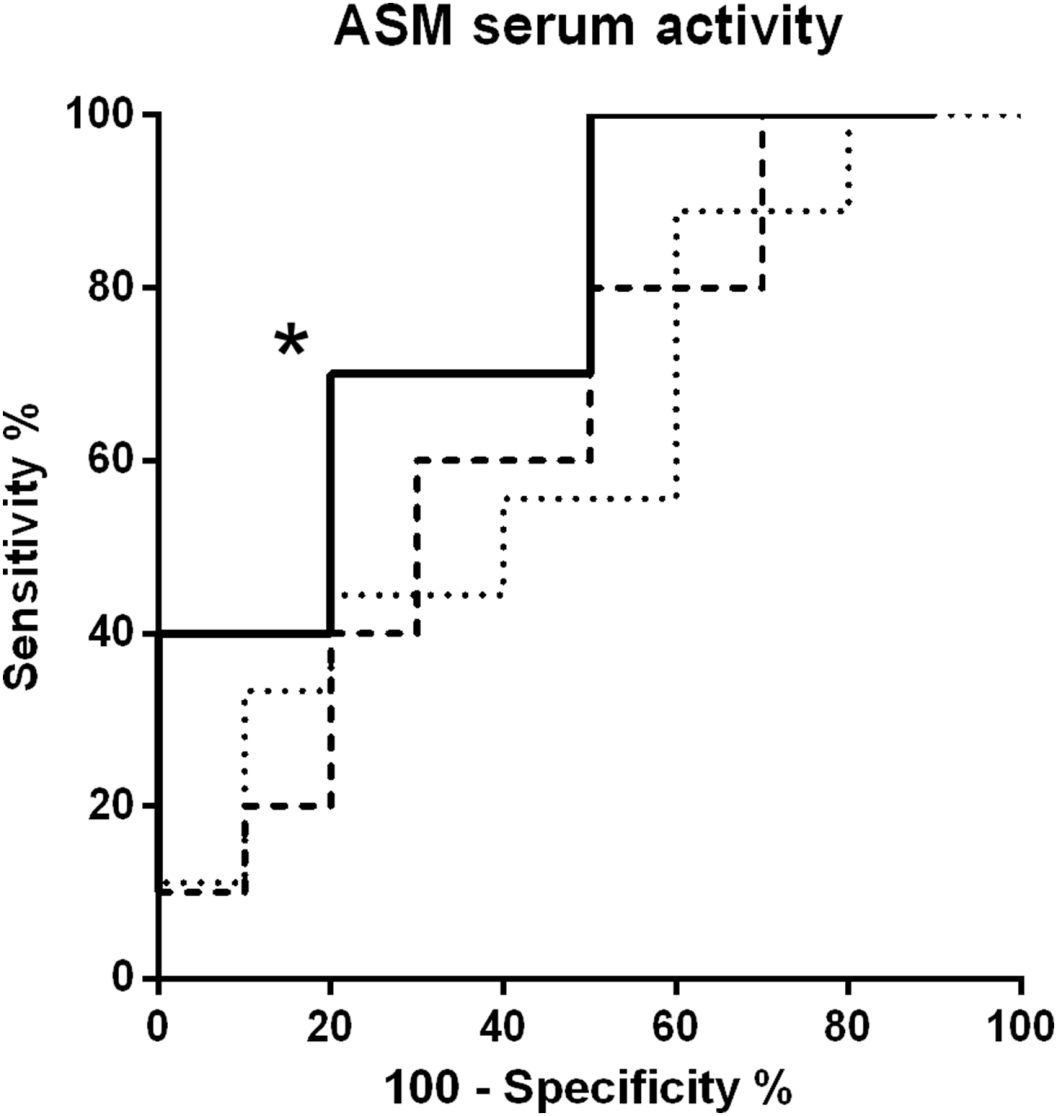
Receiver operating characteristics curve (ROC) analysis of acid sphingomyelinase serum activity. ROC analyses were performed at all three time points: *PCT_before_* (fine dashed line), *PCT_peak_* (dashed line), and *PCT_low_* (solid line). ROC curves statistically significant different from 0.5 are marked with an asterisk (*). ASM: acid sphingomyelinase serum activity.

We were also able to show the potential of ASM as prognostic marker for mortality. Although we could not detect an association to severity of illness measures, ASM remained elevated in non-surviving patients while PCT and lactate levels were lowered or within the normal range again. ROC analysis revealed a discriminative power for ASM in predicting ICU mortality at *PCT_low_* (see figure 5). It is not clear whether ASM is useful in guiding risk stratification of the critically ill patient, as an AUC of 0.79 indicates that sensitivity and specifity are not in a range where a clinically reliable discrimination between survivors and non-survivors can be derived. Other pro- and anti-inflammatory cytokines like Interleukin [IL]-10, IL-8, IL-6, IL-1β, or Tumor necrosis factor-α were shown to hold similar AUC [21]. Thus, it is most likely that the one particular biomarker for predicting outcome of patients with systemic inflammation may not exist due to the complexity of the immune response. One may speculate that ASM increases as a result of inflammation, as various inflammatory stimuli lead to activation of ASM mediated lipid signalling, including oxidative stress [22], [23], induction by or of cytokines [24], [25], platelet activating-factor (PAF)-mediated pulmonary oedema formation during acute lung injury [26] and ceramide accumulation in ischemia/reperfusion injury in mitochondria [27]. Due to the various pathophysiological properties of ASM in mediation of inflammation and apoptosis, we may also speculate that non-declining or continuously increased ASM in critically ill patients with systemic inflammation plays a putative causative role with respect to adverse outcome.

Our study has some limitations which have to be mentioned. The main limitation is the low number of patients included and that it was conducted as a single center study. Therefore, our results should be interpreted with caution as there is a high likelihood of a type II error. However, this study should be regarded in the context of a single center, pilot study aiming to gain more insight into the clinical usefulness of ASM kinetics in the course of systemic inflammation. A further limitation is that we did not perform consecutive ASM measurements in the ICU patients without systemic inflammation. This was due to financial restraints of this pilot study. Measurements of ASM are not yet comparable with established laboratory for PCT or CRP that are easier and cheaper to conduct. It is also likely that the time interval at which ASM was measured did not accurately capture an early change in ASM kinetics (24 hours range between measurement at *PCT_before_* and *PCT_peak_*). Future studies on ASM kinetics may address this potential limitation choosing an even closer measurement interval. Lastly, the potential of using ASM as a prognostic marker after systemic inflammation in clinical practice warrants further improvement of the assay to become more widely available.

## Conclusions

Our study showed that patients who underwent uncomplicated surgery exhibited a significant post-operative increase in ASM. In a mixed ICU population, ASM was significantly higher compared to these patients. While ASM did not indicate the onset of systemic inflammation earlier than PCT in our group of patients, it was able to predict ICU mortality in patients after systemic inflammation in the presence of low PCT level. ASM may hold potential as a tool for risk stratification of these patients, but the clinical value has to be further evaluated in larger studies.

## Supporting Information

File S1 **Table S1,** Procalcitonin (ng/ml) values of the included patients, sorted by survivors and non-survivors. Time points were the day before the PCT peak (*PCT_before_*), the day of the PCT peak (*PCT_peak_*) and again lowered PCT (*PCT_low_*). **Table S2,** Acid sphingomyelinase (pMol/ml*h) values for the included patients, sorted by survivors and non-survivors. Time points were the day before the PCT peak (*PCT_before_*), the day of the PCT peak (*PCT_peak_*) and again lowered PCT (*PCT_low_*). **Table S3,** C-reactive protein (mg/L) values of the included patients, sorted by survivors and non-survivors. Time points were the day before the PCT peak (*PCT_before_*), the day of the PCT peak (*PCT_peak_*) and again lowered PCT (*PCT_low_*). **Table S4,** Lactate (mMol/L) values of the included patients, sorted by survivors and non-survivors. Time points were the day before the PCT peak (*PCT_before_*), the day of the PCT peak (*PCT_peak_*) and again lowered PCT (*PCT_low_*). **Table S5,** Modified SOFA-Score (points) values of the included patients, sorted by survivors and non-survivors. Time points were the day before the PCT peak (*PCT_before_*), the day of the PCT peak (*PCT_peak_*) and again lowered PCT (*PCT_low_*). **Table S6,** SAPS-Score (points) values of the included patients, sorted by survivors and non-survivors. Time points were the day before the PCT peak (*PCT_before_*), the day of the PCT peak (*PCT_peak_*) and again lowered PCT (*PCT_low_*). **Table S7,** TISS-Score (points) values of the included patients, sorted by survivors and non-survivors. Time points were the day before the PCT peak (*PCT_before_*), the day of the PCT peak (*PCT_peak_*) and again lowered PCT (*PCT_low_*). **Table S8,** Acid sphingomyelinase activity, Procalcitonin (PCT), C-reactive protein (CRP), Lactate, modified SOFA-Score (mSofa), SAPS II Score and TISS Score of the included patients on the day of intensive care unit (ICU) admission. **Table S9,** Patients’ characteristics and outcomes of the included patients. **Table S10,** Patients’ characteristics and outcomes of included patients with systemic inflammatory response syndrome (SIRS) plus Procalcitonin (PCT) peak. **Table S11,** Patients’ characteristics and outcomes of included patients without systemic inflammatory response syndrome (SIRS) plus Procalcitonin (PCT) peak.
(PDF)
