# Supplementary material for: Acid Sphingomyelinase Serum Activity Predicts Mortality in Intensive Care Unit Patients after Systemic Inflammation: A Prospective Cohort Study
Source: PLoS One. 2014 Nov 10;9(11):e112323. doi: 10.1371/journal.pone.0112323 (PMC4226549; doi:10.1371/journal.pone.0112323)
Supplement: File S1 — Table S1, Procalcitonin (ng/ml) values of the included patients, sorted by survivors and non-survivors. Time points were the day before the PCT peak (PCTbefore), the day of the PCT peak (PCTpeak) and again lowered PCT (PCTlow). Table S2, Acid sphingomyelinase (pMol/ml*h) values for the included patients, sorted by survivors and non-survivors. Time points were the day before the PCT peak (PCTbefore), the day of the PCT peak (PCTpeak) and again lowered PCT (PCTlow). Table S3, C-reactive protein (mg/L) values of the included patients, sorted by survivors and non-survivors. Time points were the day before the PCT peak (PCTbefore), the day of the PCT peak (PCTpeak) and again lowered PCT (PCTlow). Table S4, Lactate (mMol/L) values of the included patients, sorted by survivors and non-survivors. Time points were the day before the PCT peak (PCTbefore), the day of the PCT peak (PCTpeak) and again lowered PCT (PCTlow). Table S5, Modified SOFA-Score (points) values of the included patients, sorted by survivors and non-survivors. Time points were the day before the PCT peak (PCTbefore), the day of the PCT peak (PCTpeak) and again lowered PCT (PCTlow). Table S6, SAPS-Score (points) values of the included patients, sorted by survivors and non-survivors. Time points were the day before the PCT peak (PCTbefore), the day of the PCT peak (PCTpeak) and again lowered PCT (PCTlow). Table S7, TISS-Score (points) values of the included patients, sorted by survivors and non-survivors. Time points were the day before the PCT peak (PCTbefore), the day of the PCT peak (PCTpeak) and again lowered PCT (PCTlow). Table S8, Acid sphingomyelinase activity, Procalcitonin (PCT), C-reactive protein (CRP), Lactate, modified SOFA-Score (mSofa), SAPS II Score and TISS Score of the included patients on the day of intensive care unit (ICU) admission. Table S9, Patients’ characteristics and outcomes of the included patients. Table S10, Patients’ characteristics and outcomes of included patients with system [file pone.0112323.s001.pdf]

Table S1: PCT (ng/ml)	All			Survivors			Non-survivors		
	PCTbefore	***PCTpeak***	***PCTlow***	***PCTbefore***	***PCTpeak***	***PCTlow***	***PCTbefore***	***PCTpeak***	***PCTlow***
	0,5	3,49	1,04	0,37	2,03	0,45	0,5	3,49	1,04
	0,79	28,31	0,69	2,59	7,84	1,41	0,79	28,31	0,69
	0,46	4,46	1,1	n/a	17,58	5,02	0,46	4,46	1,1
	4,95	10,1	3,6	0,09	2,61	0,13	4,95	10,1	3,6
	2,08	4,4	1,17	0,21	2,11	0,49	2,08	4,4	1,17
	4,88	12,28	0,46	0,41	3,09	0,42	4,88	12,28	0,46
	0,31	4,58	0,49	0,29	5,18	0,45	0,31	4,58	0,49
	0,28	2,29	0,38	0,17	2,52	0,39	0,28	2,29	0,38
	0,46	2,98	0,38	2,34	32,99	0,44	0,46	2,98	0,38
	1,01	2,22	0,48	0,37	100,55	0,48	1,01	2,22	0,48
	0,37	2,03	0,45						
	2,59	7,84	1,41						
	n/a	17,58	5,02						
	0,09	2,61	0,13						
	0,21	2,11	0,49						
	0,41	3,09	0,42						
	0,29	5,18	0,45						
	0,17	2,52	0,39						
	2,34	32,99	0,44						
	0,37	100,55	0,48						

Table S2: ASM (pMol/ml*h)		All			Survivors			Non-survivors	
	PCTbefore	***PCTpeak***	***PCTlow***	***PCTbefore***	***PCTpeak***	***PCTlow***	***PCTbefore***	***PCTpeak***	***PCTlow***
	4367,78	5827,1	4335,739	1833,288	1547,865	1373,608	4367,78	5827,1	4335,739
	2373,48	1911,83	2257,718	3041,235	2531,009	2185,329	2373,48	1911,83	2257,718
	1792,816	1835,107	2290	n/a	3646,628	2715,091	1792,816	1835,107	2290
	1990,827	2012,268	2167,391	3158,239	4571,43	2227,68	1990,827	2012,268	2167,391
	5569,206	3443,251	2263,291	712,98	1906,784	1653,654	5569,206	3443,251	2263,291
	2685,223	4330,894	2028,427	1836,618	1967,895	1422,425	2685,223	4330,894	2028,427
	2966,605	4266,724	2932,136	2776,618	3520,356	2830,219	2966,605	4266,724	2932,136
	3902,545	4886,777	4600,802	4927,735	1967,895	2422,425	3902,545	4886,777	4600,802
	2781,508	3022,826	3465,657	3329,174	2470,006	1975,337	2781,508	3022,826	3465,657
	5010,425	6833,413	4751,255	2307,531	4581,62	2224,006	5010,425	6833,413	4751,255
	1833,288	1547,865	1373,608						
	3041,235	2531,009	2185,329						
	n/a	3646,628	2715,091						
	3158,239	4571,43	2227,68						
	712,98	1906,784	1653,654						
	1836,618	1967,895	1422,425						
	2776,618	3520,356	2830,219						
	4927,735	1967,895	2422,425						
	3329,174	2470,006	1975,337						
	2307,531	4581,62	2224,006						

Table S3: CRP (mg/l)

All			Survivors			Non-survivors		
PCTbefore	***PCTpeak***	***PCTlow***	***PCTbefore***	***PCTpeak***	***PCTlow***	***PCTbefore***	***PCTpeak***	***PCTlow***
n/a	306,9	276,6	17,9	32,5	52,4	n/a	306,9	276,6
n/a	88,2	151,5	298,8	104	200,5	n/a	88,2	151,5
68	264	69,5	107	92,3	76	68	264	69,5
316,9	301,2	241,2	218,9	234,3	63,6	316,9	301,2	241,2
221,2	203,6	203,6	2,7	31,6	79,2	221,2	203,6	203,6
127,2	83,7	78,2	133,5	215,6	58,7	127,2	83,7	78,2
128	159,4	123	181,7	117,5	117,2	128	159,4	123
61	118	78,2	202,2	245	286,5	61	118	78,2
115,1	118,4	130,7	93,5	130,9	64,9	115,1	118,4	130,7
237	292,2	223,4	207,2	260	16,2	237	292,2	223,4
17,9	32,5	52,4						
298,8	104	200,5						
107	92,3	76						
218,9	234,3	63,6						
2,7	31,6	79,2						
133,5	215,6	58,7						
181,7	117,5	117,2						
202,2	245	286,5						
93,5	130,9	64,9						
207,2	260	16,2						

Tabel S4: Lactate (mMol/l)

All			Survivors			Non-survivors		
PCTbefore	***PCTpeak***	***PCTlow***	***PCTbefore***	***PCTpeak***	***PCTlow***	***PCTbefore***	***PCTpeak***	***PCTlow***
4,9	3,5	2,3	0,7	0,4	0,5	4,9	3,5	2,3
6,7	5,5	1	9,1	3,2	1,2	6,7	5,5	1
1,4	1	1,4	1,6	1,2	0,6	1,4	1	1,4
2,2	1,9	2,4	0,9	0,9	1,9	2,2	1,9	2,4
1,1	1	1	4,1	3,2	1,1	1,1	1	1
1,3	1,1	0,9	2	1,7	1	1,3	1,1	0,9
0,8	1	1,2	1,6	1,1	1,6	0,8	1	1,2
2,5	1,6	0,9	1,2	1,5	1,6	2,5	1,6	0,9
4,4	5,1	2,4	0,9	1,3	0,9	4,4	5,1	2,4
1,4	2,27	0,8	2,7	3,8	1	1,4	2,27	0,8
0,7	0,4	0,5						
9,1	3,2	1,2						
1,6	1,2	0,6						
0,9	0,9	1,9						
4,1	3,2	1,1						
2	1,7	1						
1,6	1,1	1,6						
1,2	1,5	1,6						
0,9	1,3	0,9						
2,7	3,8	1						

Table S5: mSOFA (points)

All			Survivors			Non-survivors		
PCTbefore	***PCTpeak***	***PCTlow***	***PCTbefore***	***PCTpeak***	***PCTlow***	***PCTbefore***	***PCTpeak***	***PCTlow***
4	6	3	9	9	5	4	6	3
10	10	3	12	13	7	10	10	3
8	8	0	6	8	4	8	8	0
6	3	3	7	8	0	6	3	3
13	13	4	14	15	3	13	13	4
2	2	1	3	3	3	2	2	1
13	13	4	7	6	1	13	13	4
1	1	1	9	9	8	1	1	1
7	6	6	1	1	7	7	6	6
6	6	6	11	12	4	6	6	6
9	9	5	6	7	3			
12	13	7						
6	8	4						
7	8	0						
14	15	3						
3	3	3						
7	6	1						
9	9	8						
1	1	7						
11	12	4						
6	7	3						

Table S6: SAPS (points)

All			Survivors			Non-survivors		
PCTbefore	***PCTpeak***	***PCTlow***	***PCTbefore***	***PCTpeak***	***PCTlow***	***PCTbefore***	***PCTpeak***	***PCTlow***
38	44	26	38	44	26	9	13	46
54	44	41	54	44	41	50	42	22
37	30	27	37	30	27	59	60	38
48	43	33	48	43	33	66	60	61
36	32	36	36	32	36	49	52	52
37	42	42	37	42	42	5	5	38
48	49	45	48	49	45	54	57	48
43	43	42	43	43	42	48	44	38
49	53	48	49	53	48	48	48	57
49	45	34	49	45	34	35	35	40
9	13	46						
50	42	22						
59	60	38						
66	60	61						
49	52	52						
5	5	38						
54	57	48						
48	44	38						
48	48	57						
35	35	40						

Table S7: TISS (points)

All			Survivors			Non-survivors		
PCTbefore	*PCTpeak*	*PCTlow*	*PCTbefore*	*PCTpeak*	*PCTlow*	*PCTbefore*	*PCTpeak*	*PCTlow*
18	10	5	18	10	5	17	25	32
23	22	18	23	22	18	30	21	21
22	18	16	22	18	16	21	18	0
23	26	26	23	26	26	13	21	31
17	18	15	17	18	15	18	8	8
20	15	13	20	15	13	8	8	0
27	18	10	27	18	10	31	23	18
9	15	5	9	15	5	15	5	0
21	21	23	21	21	23	23	15	24
24	27	18	24	27	18	31	27	18
17	25	32						
30	21	21						
21	18	0						
13	21	31						
18	8	8						
8	8	0						
31	23	18						
15	5	0						
23	15	24						
31	27	18						

Table S8: ICU admission

Patient number	***ASM***	***PCT***	***CRP***	***Lactate***	***mSOFA***	***SAPS II***	***TISS***
1	2031	29,39	446	1,6	9	63	28
2	2289	2,5	34,9	4	9	56	24
3	1466	1,1	120,9	0,5	4	16	0
4	1897	3,21	17,2	1,7	5	36	23
5	6238	2,92	59,4	1,1	3	34	4
6	2101	10,46	36,8	1,1	7	41	14
7	4060	10,14	388,8	1,2	9	63	32
8	39,4	1327	270,8	1,1	6	19	0
9	2593	0,95	130,1	0,9	2	46	19
10	4804	1,03	52,9	2,3	4	51	27
11	7199	248,73	314,3	7,6	6	62	13
12	5253	10,11	400	1,7	8	45	31
13	2071	1,26	36,6	3	7	61	22
14	3042	185,6	333,3	2,2	9	50	24
15	2464	0,3	35,7	0,9	9	46	28
16	2701	6,83	274,7	1,7	5	33	17
17	2404	2,87	7,4	1,7	8	53	15
18	2903	3,72	274,4	2,5	1	46	19
19	1690	4,61	24,5	10	4	83	24
20	n/a	3,14	34	2,1	6	16	19
21	5241	8,15	12,2	2,5	10	59	22
22	1871	1,19	168,8	0,8	4	36	10
23	2321	25,67	346,6	1,3	6	49	15
24	1532	3,79	118,3	2,1	12	53	10
25	5984	12,75	297,1	1,8	8	72	19
26	2755	4,39	9,7	0,6	4	17	10
27	1171	0,59	2,8	6,2	4	38	14
28	5569	4,88	5,6	12,7	6	75	44
29	2021	0,75	171,8	0,7	7	57	13
30	2307	0,37	201	2,7	7	38	10
31	5735	5,81	46,6	2,6	9	47	36
32	2149	0,37	239,7	0,7	8	52	19
33	4396	3,19	88,9	1,4	8	34	14

34	2965	0,14	6,6	2,8	2	34	14
35	3119	2,68	62,6	0,7	8	66	35
36	5010	1,01	234,5	1,3	7	25	19
37	1704	0,9	152,7	1,6	6	33	19
38	2908	1,57	325,6	0,9	10	41	20
39	2885	2,18	12,4	2,1	4	46	10
40	1897	5,8	155,9	0,8	6	57	10

Table S9: All Patients

Patient (no.)	Age (years)	Gender	Outcome	Reason for ICU admission
1	72	female	death	Ileus
2	86	male	death	Thoracic surgery
3	77	male	discharged	Major trauma
4	73	male	discharged	Pneumonia
5	52	male	death	Thoracic surgery
6	64	male	death	Pankreatitis
7	25	male	discharged	Pneumonia
8	73	female	discharged	Thoracic surgery
9	40	male	discharged	Pneumonia
10	50	female	discharged	Abdominal surgery
11	83	male	death	Major trauma
12	27	female	discharged	Abdominal surgery
13	54	female	death	Major bleeding
14	69	male	discharged	Soft tissue infection
15	58	male	discharged	Abdominal surgery
16	74	male	discharged	Thoracic surgery
17	66	male	discharged	Thoracic surgery
18	72	female	discharged	Mesenterial ischaemia
19	43	male	discharged	Major trauma
20	77	male	discharged	Thoracic surgery
21	69	female	discharged	Abdominal surgery
22	35	male	discharged	Abdominal surgery
23	76	female	death	Abdominal surgery
24	81	male	death	Mesenterial ischaemia
25	46	male	discharged	Pneumonia
26	73	female	discharged	Soft tissue infection
27	32	male	death	Liver failure
28	58	male	death	Abdominal surgery
29	89	female	death	Abdominal surgery
30	80	male	discharged	Pneumonia
31	70	female	death	Abdominal surgery
32	63	male	discharged	Soft tissue infection
33	67	male	discharged	Abdominal surgery

34	71	female	discharged	Pancreatitis
35	77	female	death	Abdominal surgery
36	44	male	discharged	Abdominal surgery
37	88	male	discharged	Pneumonia
38	57	male	death	Abdominal surgery
39	82	female	death	Major bleeding
40	68	male	discharged	Thoracic surgery

Table S10: ICU patients with SIRS plus PCT peak

Patient (no.)	Age (years)	Gender	Outcome	Reason for ICU admission
23	76	female	death	Abdominal surgery
28	58	male	death	Abdominal surgery
31	70	female	death	Abdominal surgery
35	77	female	death	Abdominal surgery
27	32	male	death	Liver failure
11	83	male	death	Major trauma
6	64	male	death	Pancreatitis
2	86	male	death	Thoracic surgery
5	52	male	death	Thoracic surgery
10	50	female	discharged	Abdominal surgery
21	69	female	discharged	Abdominal surgery
33	67	male	discharged	Abdominal surgery
36	44	male	discharged	Abdominal surgery
34	71	female	discharged	Pancreatitis
25	46	male	discharged	Pneumonia
37	88	male	discharged	Pneumonia
8	73	female	discharged	Thoracic surgery
17	66	male	discharged	Thoracic surgery
20	77	male	discharged	Thoracic surgery

Table S11: ICU patients without SIRS plus PCT peak

Patient (no.)	Age (years)	Gender	Outcome	Reason for ICU admission
12	27	female	discharged	Abdominal surgery
15	58	male	discharged	Abdominal surgery
22	35	male	discharged	Abdominal surgery

29	89	female	death	Abdominal surgery
38	57	male	death	Abdominal surgery
1	72	female	death	Ileus
13	54	female	death	Major bleeding
39	82	female	death	Major bleeding
3	77	male	discharged	Major trauma
19	43	male	discharged	Major trauma
18	72	female	discharged	Mesenterial ischaemia
24	81	male	death	Mesenterial ischaemia
4	73	male	discharged	Pneumonia
7	25	male	discharged	Pneumonia
9	40	male	discharged	Pneumonia
30	80	male	discharged	Pneumonia
14	69	male	discharged	Soft tissue infection
26	73	female	discharged	Soft tissue infection
32	63	male	discharged	Soft tissue infection
16	74	male	discharged	Thoracic surgery
40	68	male	discharged	Thoracic surgery

Table S12: Control group

Patient (no.)	Age (years)	Gender	Outcome	Reason for ICU admission
1	62	female	discharged	Abdominal surgery
2	71	female	discharged	Abdominal surgery
3	69	male	discharged	Abdominal surgery
4	69	male	discharged	Abdominal surgery
5	58	male	discharged	Abdominal surgery
6	92	female	discharged	Abdominal surgery
7	58	male	discharged	Abdominal surgery
8	46	male	discharged	Abdominal surgery
